# Liming enhances longevity of wheat seeds produced in acid soils

**DOI:** 10.1038/s41598-022-21176-6

**Published:** 2022-10-27

**Authors:** Tiago Alexandre da Silva, Carlos Alexandre Costa Crusciol, Thiago Barbosa Batista, João William Bossolani, Gustavo Roberto Fonseca de Oliveira, Denise Puntel Basso, Antônio Carlos de Almeida Carmeis Filho, Juliana Pereira Bravo, Edvaldo Aparecido Amaral da Silva

**Affiliations:** Department of Crop Science, College of Agricultural Sciences, São Paulo State University, Botucatu, Brazil, Sao Paulo State University (UNESP), 237 Botucatu, São Paulo, CEP 18610-307 Brazil

**Keywords:** Plant sciences, Plant molecular biology

## Abstract

The environment where plants grow, such as acidic soils, interferes with the nutrient concentration and physiological quality of seeds. This hypothesis was tested using wheat seeds as a model crop, grown in a tropical soil with and without lime application for twelve years. Here we show that lime provides remarkable enhancements in soil chemistry and seed composition, without altering the seed’s germination and vigor. Also, it favors the production of seeds with additional molecular mechanisms that extend their longevity. Our results indicate that the application of lime mitigates acidity in tropical soils and ensures the production of seeds with enhanced chemical composition and longer life span.

## Introduction

Wheat is an important cereal produced worldwide. Its propagation is carried out by seeds. Thus, it is desirable that they exhibit high physiological performance, which involves a complex network of attributes that reflect adequate germination, vigor, and storage capacity (longevity) to ensure the establishment of the seedling.

Longevity is the ability of a seed to remain viable during storage^1^. Multiple mechanisms are involved with this attribute, such as the non-reducing sugars, late embryogenesis abundant proteins (LEA), RNA binding proteins that conserve seed mRNA in the dry state, antioxidant molecules, and heat shock proteins (HSPs)^2,3^. The reports of seed longevity have focused on understanding the underlying mechanisms that govern this important attribute^4,5^. This is especially true because seed longevity is strongly impacted by stressful conditions experienced by the mother plant during seed maturation^4,6^.

In tropical environments, acid soils are considered a stressful condition to plants since they reduce the availability of nutrients, such as calcium, magnesium and phosphorus, and increase the bioavailability of toxic elements (aluminum and iron); which reduces the grain yield of crops with agronomic interest^7,8^. Due to these detrimental effects, lime is commonly applied to acid soils to increase the yield potential of crops, as it increases the soil pH and base saturation, which favors the availability of nutrients for plants, and reduces the toxicity of Al^3+^ and Fe^9^. It was demonstrated that acid soils lead to a reduction in the nutritional status of wheat leaves, and the application of lime increased the grain yield in wheat plants^10^.

Nonetheless, the impact of acid soils and the consequence of liming on the seed physiological potential is little understood in wheat seeds. It is known that the transference of the nutrients from the plant to the seed is required during development. It was demonstrated^11^ that barley seeds produced in an environment without an adequate supply of nutrients in the soil are more sensitive to artificial aging. Thus, chemical components in the soil can impact seed storability. Based on this, we hypothesized that changes in the nutrient status of the seeds produced in acid soils has an influence on seed storability. To study this, we investigated if physiological aspects and nutritional status of wheat seeds produced in acid soils with and without liming management, influences seed longevity.

## Materials and methods

### Plant material and growth conditions

This study used seeds produced in a long-term experimental field located in Botucatu, São Paulo State, Brazil (48°23′ W, 22°51′ S, altitude of 765 m) (registered on the GLTEN Metadata Portal; https://www.glten.org/experiments/62). The data presented correspond to wheat crops cultivated in 2013 and 2014. According to the Köppen-Geiger climate classification system^12^, the climate in the region is Cwa, characterized as mesothermic type, with dry winters and hot summers. The soil is classified as a sandy clay loam kaolinitic thermic Typic Haplorthox^13^. Before establishing the experiment (October 2002), the soil fertility and texture were determined at 0.0–0.20 m, according to the methodology described by^14^ as follows: soil organic matter (SOM): 20.9 g kg^−1^; pHCaCl_2_: 4.2; P_resin_: 9.2 g kg^−1^; exchangeable Al^3+^, K^+^, Ca^2+^, and Mg^2+^: 2.3, 1.2, 14.0 and 5.0 mmol_c_ kg^−1^, respectively; total acidity in pH 7.0 (H + Al): 37 mmol_c_ kg^−1^, cation exchange capacity (CEC): 58 mmol_c_ kg^−1^; sand, silt, and clay content: 545, 108, and 347 g kg^−1^, respectively. In general, this soil is typical of acidic Oxisols found in Brazil.

Over the 12-year experimental period, the lime treatments were applied three times (October 2002, 2004 and 2010). The seeds of this study were colected in the third and fourth year after the last lime reapplication (2010). Details of previous crops and treatments are described in Supplementary Table S1.

The experiment design was a randomized complete block with four replications. The treatments consisted of two lime levels applied to the soil surface (without incorporation) as follows: no-lime and 2000 kg ha^−1^ (full-recommended rate) of dolomitic lime. The full recommended dolomitic lime rate (DR) was calculated to increase the base saturation in the topsoil layer (0.0–0.20 m) to 70% according to the methodology proposed by^15^ (Supplementary Table S2). In all applications, dolomitic lime acted as liming agent; its composition was 233 g kg^−1^ of CaO, 175 g kg^−1^ of MgO, and 88% of effective calcium carbonate equivalent.

Wheat (cv. CD116) was sown in April 18th, 2013 and March 12th, 2014, at a density of ~ 20 viable seeds m^−1^ at rows spaced at 0.17 m apart. The model species in this research belongs to a commercial cultivar commonly grown in Brazil. Therefore, it complies with the relevant institutional, national, and international guidelines and legislation regarding the use of biological material. Soil fertilization was carried out according to the soil chemical characteristics determined previously at the beginning of the crop season. Mean temperature and rainfall during the period of wheat seed production were 19 °C and 264 mm in 2013; 21 °C and 280 mm in 2014, respectively (suplemantary Fig. S1). The wheat harvest occurred on August 22nd, 2013 and June 24th, 2014. The harvest was performed mechanically and the seeds were stored in a cold chamber with a temperature of 12 °C and relative humidity of 50%. In this condition, water content of the seeds was ~ 11.7% (g kg^−1^ of water) for the no-lime treatment, and ~ 10.9% for the 2000 kg ha^−1^ lime treatment. We determined the water content by the oven method at 105 ± 3 °C for 24 h, and the results were expressed as wet basis.

### Physiological performance

To assess seed germination, four replicates containing 50 seeds each were germinated in rolled paper towel moistened with distilled water equivalent to 2.5 times its weight, at 20 °C in the dark. The time required for germination was determined by a daily count of the number of seeds with radicles ≥ 1 mm. The t_50_ was calculated by using the cumulative curve adjustment module of the Germinator software package^16^. The germination percentage was scored at eight days from the beginning the incubation period, by counting the number of normal seedlings.

To investigate the seed longevity, we used the ageing protocol, in which the seeds were distributed in single layers on the surface of a wire mesh screen suspended over an NaCl solution (75% RH) inside a plastic box (11.0 × 1.0 × 3.5 cm) hermetically sealed, at 35 °C. The seeds were sampled at intervals of approximately 15 days, and their viability was determined as described for germination above using the radicle ≥ 1 mm as criteria. Longevity (*p*_50_) was defined as the time (number of days) at which the seeds had lost 50% of their viability during storage, and was calculated using the equation: *v* = *(K*_*i*_*−p)/σ*^17^.

### Analysis of nutrients in the seeds

The analysis of macro and micronutrients was performed according to^18^. Four samples of thirty seeds were macerated. They were digested using concentrated sulfuric acid with Na_2_SO_4_, and catalysts (CuSO_4_.5H_2_0 and Na_2_SeO_3_) to evaluate nitrogen (N) using 0.100 g of each sample. The nitric-perchloric method was used with 0.250 g of each sample to determine the amounts of P, K, Ca, Mg, S, Cu, Fe, Mn, and Zn. After extraction, the nutrients were determined by the following methods: semi-micro-Kjeldahl (N), colorimetry of metavanadate (total P), barium sulfate (S) turbidimetry and atomic absorption spectrophotometry (Ca, Mg, K, Cu, Zn, Mn, and Fe), according to^18^.

### Quantification of gene expression by real-time PCR

RNA was extracted in samples of the embryonic axis at 60 days of storage according to^19^, modified with sodium dodecyl sulfate (SDS)/TRIzol, with the extraction buffer consisting of 100 mM Tris–HCl (pH 9.0) and 2% β-mercaptoethanol (v/v), prepared with MilliQ autoclaved water treated with DEPC (diethyl pyrocarbonate). We used three biological samples from the control group (without liming) and three from the group that received the recommended dose (2000 kg ha^−1^). In total, 12 RNA samples were extracted from 30 embryonic regions. cDNA was synthesized from a 2-μg sample of high-quality total RNA using a High-Capacity RNA-to-cDNA kit (Applied Biosystems), following the manufacturer’s instructions. The cDNA reaction mixture consisted in 2 μL of 10X RT Random Primers, 2 μL of 10 × RT Buffer, 0.8 μL 25X dNTP Mix (100 mM), 1 μL MultiScribeTM Reverse Transcriptase, and 4.2 μL Nuclease-free H_2_O. Finally, the cDNA mixture (10 μL) was homogenized with RNA (10 μL) and taken to the thermocycler for initial enzyme activation (25 °C for 10 min), cDNA synthesis (37 °C for 120 min), inactivation of the enzyme (85 °C for 5 min) and ending the cycling under constant 4 °C.

Quantitative real-time PCR was performed in a 12-μL volume, consisting of 3 μL of cDNA solution at a concentration of 2 µg µL^−1^, 6 μl of 2 × SYBR Green qPCR ReadyMix (Sigma Aldrich), 0.5 μL of 10 mM of both reverse and forward gene-specific primer solution (10 mM), and 2.5 μL of UltraPure distilled water. The amplification was performed in an Eco Real-Time thermocycler (Illumina) with an initial 2 min incubation period at 50 °C, followed by 3 min at 95 °C, then 40 cycles of 15 s at 95 °C and 30 s at 60 °C. At the end of the reactions, a melting curve was performed, which consisted of the following steps: 15 s at 95 °C, 55 °C, and 95 °C, respectively (made by the software itself). The data were analyzed using the EcoStudy program version 5.0 (Illumina). For this assay, the genes related to longevity were screened using studies in the literature that combined consistent physiological results with differential expression and phenotyping after mutation. The sequence of genes used to design the primers was taken from the NCBI database (Table S3), for *T. aestivum* homologous sequences. Primer efficiency was calculated using the LinRegPCR program.

Relative expression levels were calculated using the comparative ^△△^(Ct) method^20^ using the reference genes ADP-ribosylation factor, RNAse 1 inhibitor-like protein, α-tubulin, and histone.

### Statistical analysis

The comparison between the groups occurred by t-test at p ≤ 0.05-confidence level. To quantify the relative gene expression, we used the REST® program to perform comparative quantification through the “Pair-Wise Fixed Reallocation Randomization Test” method^21^, comparing a group of samples with the control. For this analyze the control group refers to seed sample different from those with and without lime, used especially for this assay. Pearson correlation was performed using *p*_50_ of the wheat seeds produced without and with lime versus the amount of nutrients and gene expression.

Finally, due to dependence and interdependence of variables analyzed at 60 days after storage with seed longevity, the triplot redundancy analysis (RDA) was performed to determine the correlations among soil fertility × seed nutrients, soil fertility × longevity gene expression and *p50*, and seed nutrients × longevity gene expression and *p50*. Forward selection (FS) and Monte Carlo permutation tests were applied with 999 random permutations to verify the significance of nutrients on longevity parameters. RDA plots were generated using the Canoco 4.5 software (Biometrics, Wageningen, the Netherlands). We used the two-way PERMANOVA^22^ to group the treatments for similarity (Bray–Curtis).

## Results

### Soil chemical properties

Soil sampling was performed at 0.0–20 m depth in October 2013 (between the two crop seasons) to perform the soil chemical properties analysis and thus, to analyze the influence of soil attributes on seed quality. Based on soil fertility, we verified that long-term liming was efficient to reduce the soil acidity changing the soil pH from 4.1 to 5.2, as well as increasing the SOM content (Table 1). Consequently, increases of macronutrient concentrations (P_resin_, Ca, and Mg), and reduction in H + Al and Al concentrations also occurred in lime-amended soil compared with no-lime (control). The cascade effect provided by liming increased soil pH and basic cation concentration (especially Ca and Mg), and reduced H + Al concentration, resulting in higher base saturation. Interestingly, the concentration of Fe in seeds was reduced in the lime-amended soil, whereas the concentrations of Mn, Cu and Zn did not change.

**Table 1:** Chemical characteristics of the soil at 0–0.20 m depth in 2013 and 2014.

	pH	SOM	P_resin_	S	K	Ca	Mg
	(CaCl_2_)	(g kg^−1^)	(mg kg^−1^)		(mmol_c_ kg^−1^)		
0–0.20 m depth in 2013							
No-lime	4.1 ± 0.07b	21 ± 1.21b	23 ± 3.65b	19 ± 3.23a	2 ± 0.19a	9.3 ± 2.99b	3 ± 1.05b
Lime	5.2 ± 0.21a	23 ± 0.78a	41 ± 8.49a	18 ± 1.34a	2 ± 0.43a	35.9 ± 4.61a	14.42 ± 2.1a

	Al	H + Al	BS	Fe	Mn	Cu	Zn
	(mmol_c_ kg^−1^)		(%)	(mg kg^−1^)			
0–0.20 m depth in 2013							
No-lime	14 ± 1.48a	87 ± 4.91a	14 ± 2.95b	22 ± 0.58a	24 ± 4.36a	3 ± 0.37a	4.2 ± 0.27a
Lime	2 ± 1.25b	45 ± 4.41b	53 ± 3.85a	17 ± 1.27b	24 ± 2.5a	3 ± 0.05a	3.7 ± 0.77a

	pH	SOM	P_resin_	S	K	Ca	Mg
	(CaCl_2_)	(g kg^−1^)	(mg kg^−1^)		(mmol_c_ kg^−1^)		
0–0.20 m depth in 2014							
No-lime	4.2 ± 0.1b	21 ± 0.8b	24 ± 4.5b	15 ± 0.9b	2.1 ± 0.12a	11 ± 2.8b	3.5 ± 1.1b
Lime	5.4 ± 0.14a	24 ± 1.2a	44 ± 6.3a	18 ± 0.9a	2.3 ± 0.4a	43 ± 2.7a	16 ± 1.9a

	Al	H + Al	BS	Fe	Mn	Cu	Zn
	(mmol_c_ kg^−1^)		(%)	(mg kg^−1^)			
0–0.20 m depth in 2014							
No-lime	12 ± 1.4a	77 ± 4.08a	17 ± 0.8b	20 ± 0.8a	23 ± 2.6a	2.7 ± 2.6a	3.9 ± 0.2a
Lime	1.9 ± 0.8b	35 ± 1.5b	63 ± 2.1a	16 ± 1.1b	22 ± 2.4a	2.8 ± 0.04a	3.4 ± 0.7a

Means ± standard error followed by different letter differ by the t-test at 0.05 confidence level.

### Liming on seed physiological performance

The variation in initial seed water content was 0.8%, showing uniformity among wheat seeds produced with different levels of liming. This information allowed the comparison of the studied groups regarding their physiological performance.

**Figure 1:**
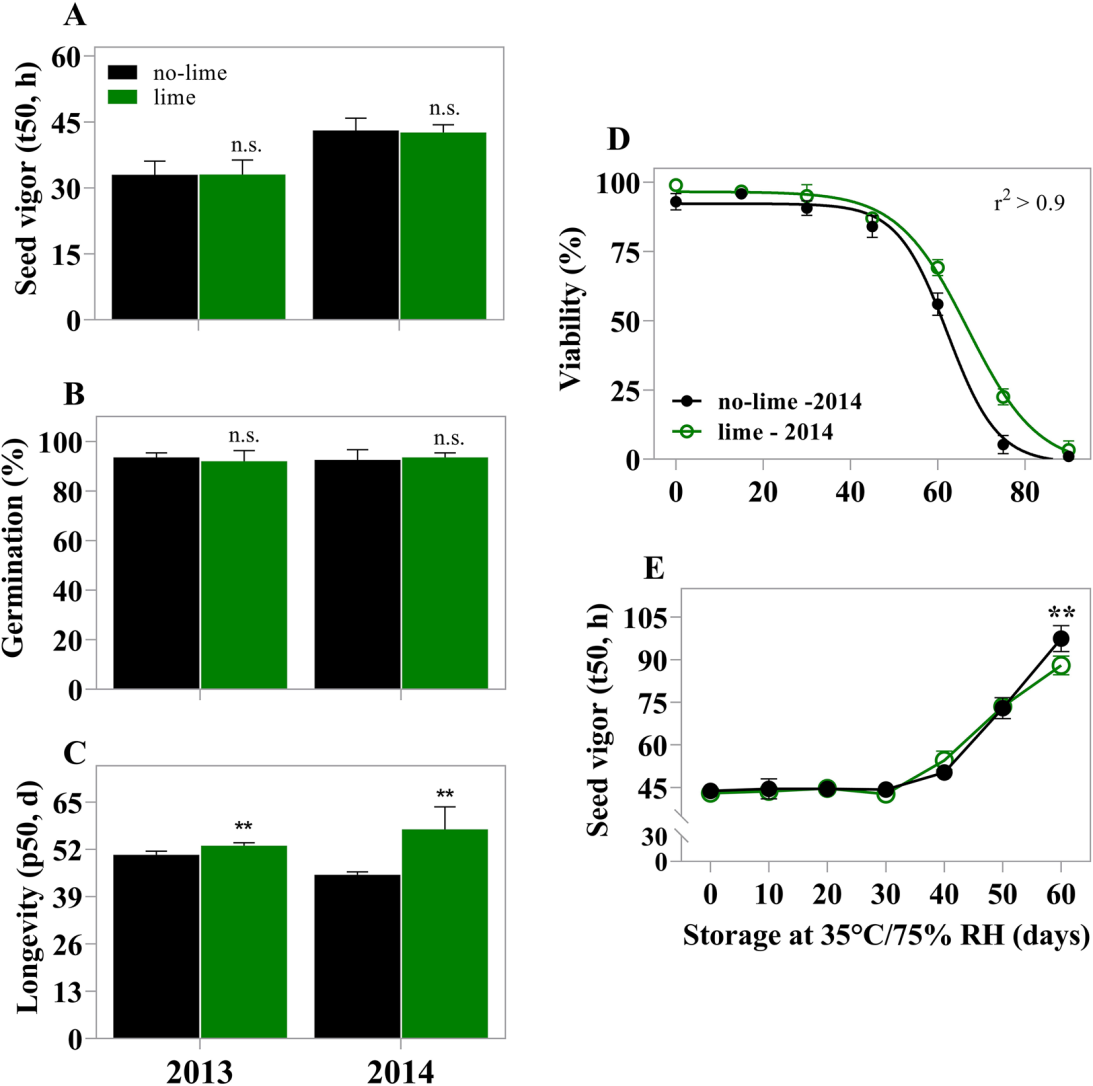
Physiological quality of wheat seeds produced without liming and with lime application at a rate of 2000 kg ha^−1^. (**A**) Seed germination of normal seedlings. (**B**) Seed vigor (t50). (**C**) Values of *p*_50_ in wheat seeds produced with and without lime. (**D**) Germination of wheat seeds from the 2014 season produced with and without lime collected at different times during storage, fitted using the Boltzmann equation parameters [*y* = top + (bottom-top)/1 + [*v*50 − (*x*/slope)]; r^2^ > 0.9 for both groups: lime and non-lime]. (**E**) Seed vigor (t50) during storage in seeds from the 2014 season produced without and with lime. Data points are averages of eight replicates of 50 seeds each, and bars represent standard deviation. The two asterisks represent the significance at 0.05-confidence level by the t test between lime and no-lime. n.s. not significative.

The germination and seed vigor (t50) did not change with liming, regardless of the crop season (Fig. 1A,B). Despite this, vigor was higher in 2014, which can be associated with several factors that favor its greater expression. These factors were not explored in this study. However, we found that liming changed the seed longevity (*p*_50_) (Fig. 1C,D). The *p*_50_ was higher in seeds produced in lime-amended soils (Fig. 1C). The *p*_50_ was 50 days for seeds that were not limed and 54 days for those that received the recommended rate (2000 kg lime ha^−1^) in the 2013 crop season. It was 45 days for seeds that were not limed and 60 days for seeds that were limed in 2014 crop season (Fig. 1C). Thus, there was a difference in seed viability during storage between wheat seeds produced in soils with and without liming in the two crop seasons.

For the seeds produced in 2014, the loss of viability during storage was adjusted to a sigmoidal behavior (r^2^ > 0.9). It was observed that liming slows down the loss of seed viability during storage (Fig. 1D). In addition, our results demonstrated that the wheat seeds produced in limed soil preserved seed vigor (t50) before the loss of 50% viability (Fig. 1E). Based on these results, we decided to investigate the influence of liming in acid soils on longevity using seeds from the 2014 crop season.

### Seed nutrition concentration

We analyzed the nutrient status of wheat seeds produced in the 2014 crop season through determinations of macro and micronutrients in these samples. Our results revealed that wheat seeds produced in lime-amended soil presented higher concentrations of N, P, K, and Mg, and a lower concentration of Mn (Table 2). The concentrations of Ca, S, Fe, Cu, and Zn did not change with liming.

**Table 2:** Nutrient concentration in wheat seeds from 2014 crop season.

Treatments	N	P	K	Ca	Mg
	(g kg^−1^)				
No-lime	12 ± 1.13b	1.84 ± 0.33b	3.7 ± 0.52b	2.8 ± 0.17a	1.6 ± 0.06b
Lime	20 ± 0.66a	3.05 ± 0.03a	5.1 ± 0.35a	3.9 ± 0.71a	2.1 ± 0.09a

Treatments	S	Fe	Mn	Cu	Zn
	(g kg^−1^)	(mg kg^−1^)			
No-lime	1.0 ± 0.05a	43.0 ± 9.77a	76.7 ± 9.04a	10.3 ± 7.42a	65.5 ± 19.87a
Lime	0.9 ± 0.04a	47.3 ± 8.40a	51.0 ± 1.71b	16.5 ± 14.03a	49.3 ± 1.5a

Means ± standard error followed by different letter differ by the t-test at 0.05 confidence level.

### Gene expression in stored seeds from soils with and without liming

**Figure 2:**
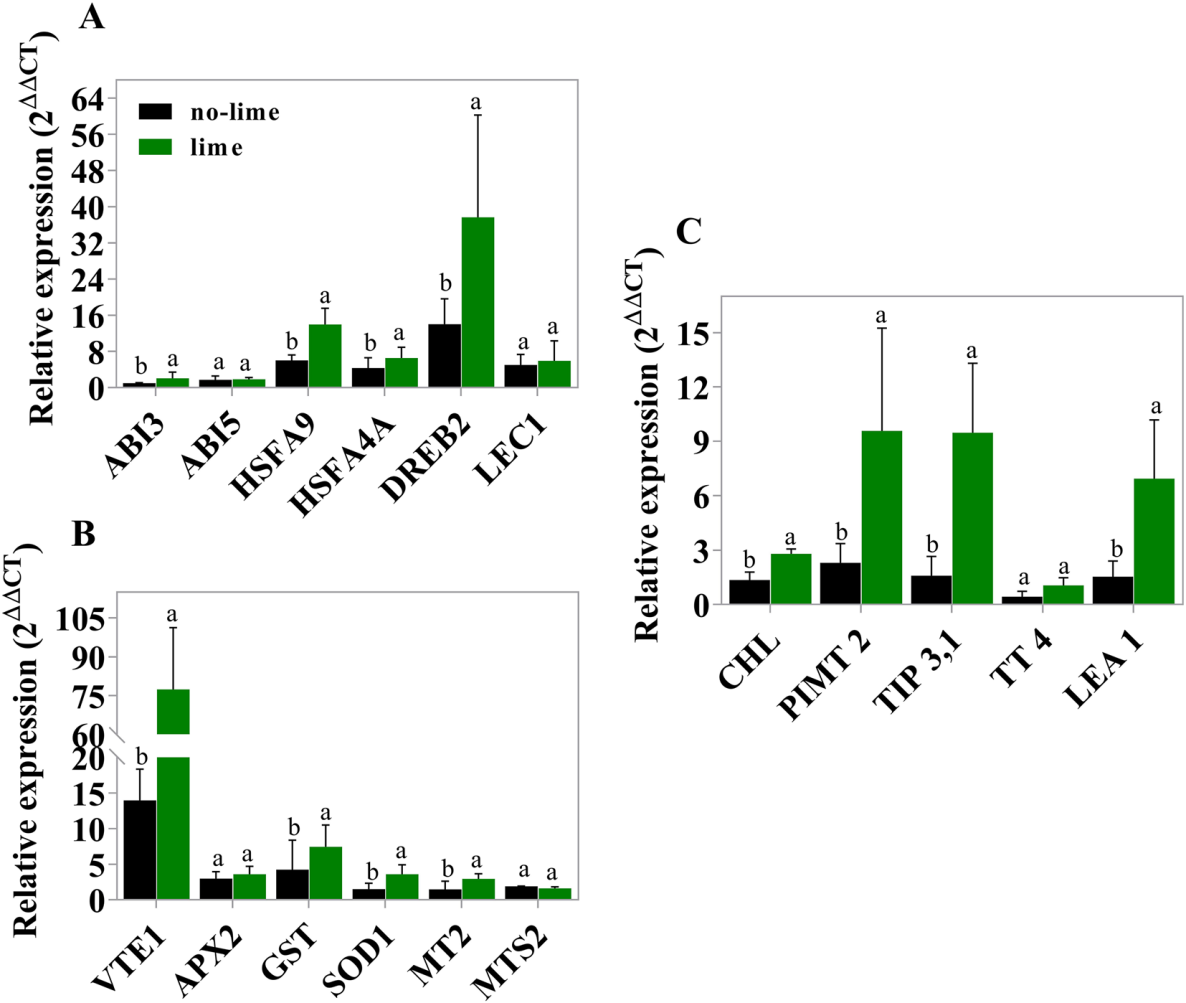
(**A**) Relative expression of transcription factors in the embryonic region of wheat seeds: ABA- insensitive 3 (*ABI3*), ABA-insensitive 5 (*ABI5*), heat shock transcription factor A9 (*HSFA9*), heat shock transcription factor A4A (*HSFA4A*), drought responsive element binding protein 2 (*DREB2*), and leafy cotyledon 1. (**B**) Relative expression of antioxidants in the embryonic region of wheat seeds with and without lime and stored for different periods of time: ascorbate peroxidase 2 (*APX2*), superoxide dismutase 1 (*SOD1*), glutathione s-transferase (*GST*), metallothionein 2 (*MT2*), mercaptopyruvate sulfur transferase 2 (*MST2*), and vitamin E deficient 1 (*VTE1*). (**C**) Relative expression of genes associated with the synthesis of proteins related to longevity in the embryonic region of wheat seeds: chloroplastic lipocalin (CHL), protein-l-isoaspartate methyltransferase 2 (PIMT 2), alpha-tonoplast intrinsic protein (*TIP3, 1*), transparent testa 4 (TT 4) and EM6 (*LEA 1*). The same lowercase letters indicate that the seeds in the treatments did not differ by the Tukey test at the 5% level of probability after 60 days of storage. Error bars indicate standard deviation (SD) of three biological replications.

We analyzed the expression of genes involved with different longevity properties (i.e., resistance to stress, oxidative stress and seed protein remodeling) of seeds previously reported in the literature^27–31^. The expression of transcription factors *ABI3*, *HSFA9*, *HSFA4A*, and *DREB2* increased during storage in seeds from plants established in lime-amended soils, while the expression of two other transcription factors (*ABI5* and *LEC1*) did not change (Fig. 2A).

The expression of genes associated with the antioxidant active system increased in seeds from soils with liming. There was a significant increase in the expression of these genes during storage, including vitamin E deficient 1 (*VTE1*), superoxide dismutase 1 (*SOD1*), glutathione s-transferase (*GST*), and metallothionein 2 (*MT2*). The expression of ascorbate peroxidase 2 (*APX2*) and mercaptopyruvate sulfurtransferase 2 (*MST2*) did not change (*p* > 0.05) during storage (Fig. 2B).

Gene expression of protein synthesis related to seed longevity, such as chloroplastic lipocalin (CHL), protein-l-isoaspartate methyltransferase 2 (*PIMT 2*), alpha-tonoplast intrinsic protein (*TIP3,1*) and *EM6-LEA 1* increased in seeds from lime-amended soils (Fig. 2C).

### Soil fertility, seed nutrient concentration and longevity plus gene expression at 60 days of storage

**Figure 3:**
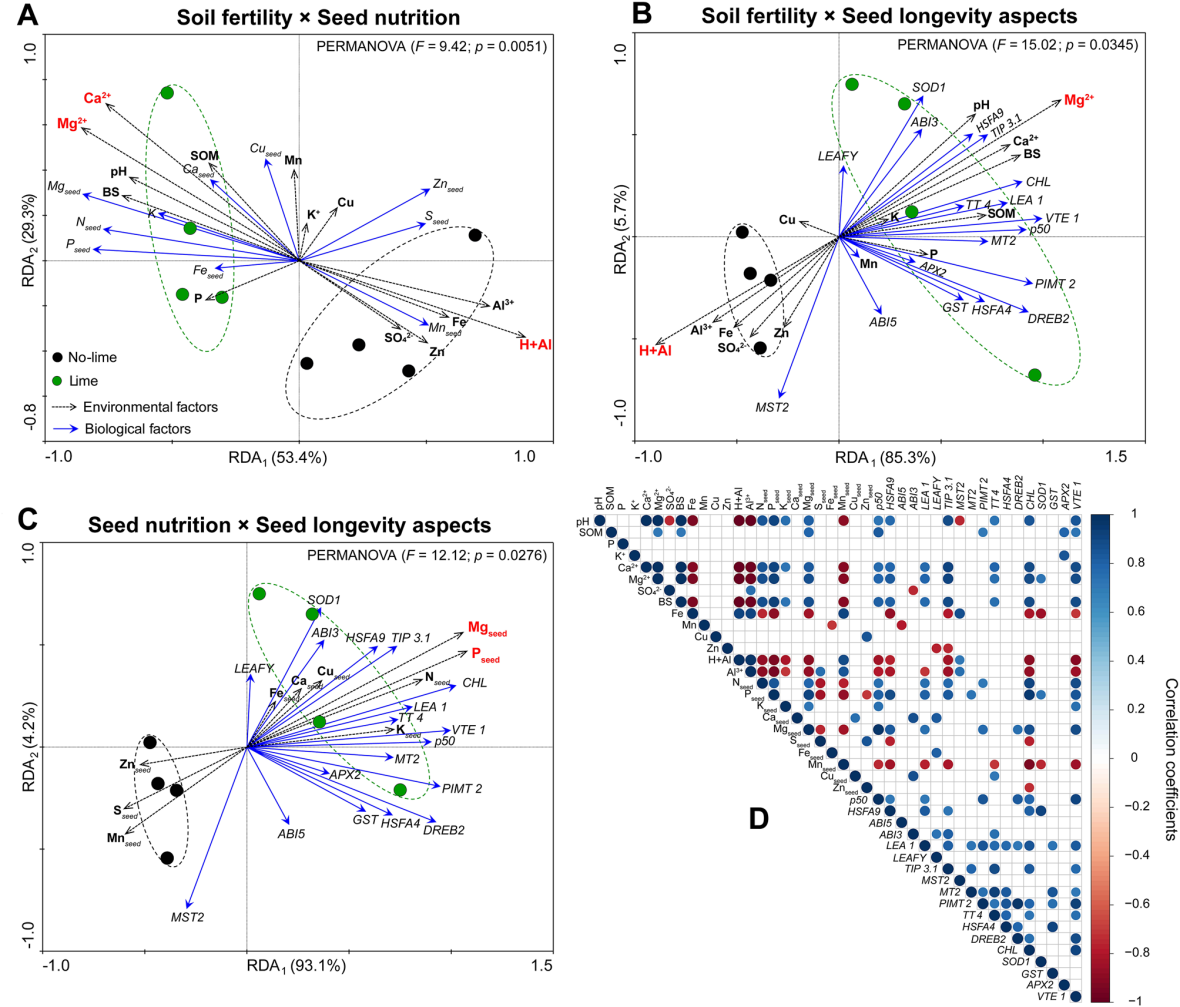
Triplot redundancy analysis (RDA) of the nutrient concentration, longevity gene expression and *p*50 of wheat seeds. Arrows indicate correlations between factors. The significances were evaluated by a Monte Carlo permutation test (999 permutations) and are indicated by the red color (*P* ≤ 0.05). The dashed lines indicate significant clusters according to the permutation analysis (PERMANOVA, *P* ≤ 0.05). Heatmap correlation (Pearson) based on the same factors used in the RDA. Only significant correlations (*P* ≤ 0.05) are shown.

Soil fertility was responsible for 53.4% and 85.3% of the total variation in seed nutrient content and longevity aspects, respectively (Fig. 3A,B). Seed nutrient concentration was responsible for 93.1% of the total variation in seed longevity aspects. In addition, PERMANOVA analysis (p < 0.05) clearly segregated treatments into 2 clusters (black = no-lime; green = lime) in the arrangements analyzed (Fig. 3A–C). Monte Carlo permutation analysis indicated Mg^2+^ and Ca^2+^ concentration in the soil was the main responsible for modulating variance in seed nutrients, and Mg for seed longevity aspects. In both cases, these nutrients were strongly related to cluster segregation with seed samples from the lime treatment. Additionally, H + Al concentration in the soil was the main factor responsible for negatively influencing seed nutrient content and longevity.

Specifically in the seeds domain, Mg and P concentrations were mainly responsible for modulating seed longevity aspects with a strong relation with seed samples from the lime treatment. Additionally, the *p*50 vector demonstrated strong proximity to the vectors of the VTE 1, TT 4, MT2, LEA 1, CHL and PIMT 2 genes.

Finally, Person’s correlations showed that the *p*50 correlated with pH, SOM, Mg^2+^, Ca^2+^ and base saturation in soil; N, P, K and Mg concentration in seeds and the expression of HSFA9, ABI5, LEA 1, PIMT 2, DREB2, CHL, and VTE1 genes in seeds (Fig. 3D).

## Discussion

Acid soils reduce the availability of elements essential for plant nutrition under tropical conditions. Liming is a common and effective practice that mitigates the nutritional deficiency in the soil from tropical areas of the globe. We show that liming influences the chemical composition of seeds and the expression of genes associated with longevity. Therefore, it can be used to ensure the production of seeds with superior storability when these are produced in tropical regions.

The change in soil chemistry under the effect of lime expanded the supply of elements likely to be taken up by wheat plants (Table 1), without, however, affecting seed germination and vigor (Fig. 1A,B). Also, soil acidity management favored the production of seeds with a superior capacity to preserve their viability and vigor during storage (Fig. 1D,E), which ensures superior longevity in relation to seeds produced in acid soils without liming (Fig. 1C). The capacity of maintaining viability during storage is associated with protection mechanisms installed during the acquisition of longevity^5^. Unlike the other physiological quality attributes, seed longevity exhibits high plasticity^2^. This means that changes in the maternal environment during seed development or during the reproductive phases can amplify or limit retention of this attribute^1,4^. In the case of seed vigor, in some plant species, environmental conditions, such as air temperature during the cycle, can also alter this physiological quality attribute^23,24^; such as observed for t50. We observed that there was a difference of two degrees from one year to the other (the average for 2013 was 19 °C and for 2014 21 °C). This factor could explain the difference observed in vigor (Fig. 1A). However, highlighting the effect of the treatments in this research, we reinforce that lime was the factor responsible for changing the growth environment (because it changed the soil chemistry from acid to non-acid) and enhancing longevity in seeds (Fig. 1E). This is a huge benefit for wheat-based food production in tropical conditions, since better quality seeds represent higher yields^25^.

Previous evidence has associated high sensitivity to aging in seeds from plants that experienced inadequate nutritional supply during their development^11,26^. We found higher concentrations of several nutrients (Table 2) and a strong positive correlation between magnesium and phosphorus with superior longevity in wheat seeds from plant grown in lime-amended soil (Fig. 3C,D). Our results in parallel with reports mentioned earlier, clarify a connection between the nutrient amounts in the soil/seeds with seed longevity (Fig. 3A–C). These findings proved our hypothesis and advanced the comprehension of the implications of soil chemistry on longevity of wheat seeds.

**Figure 4:**
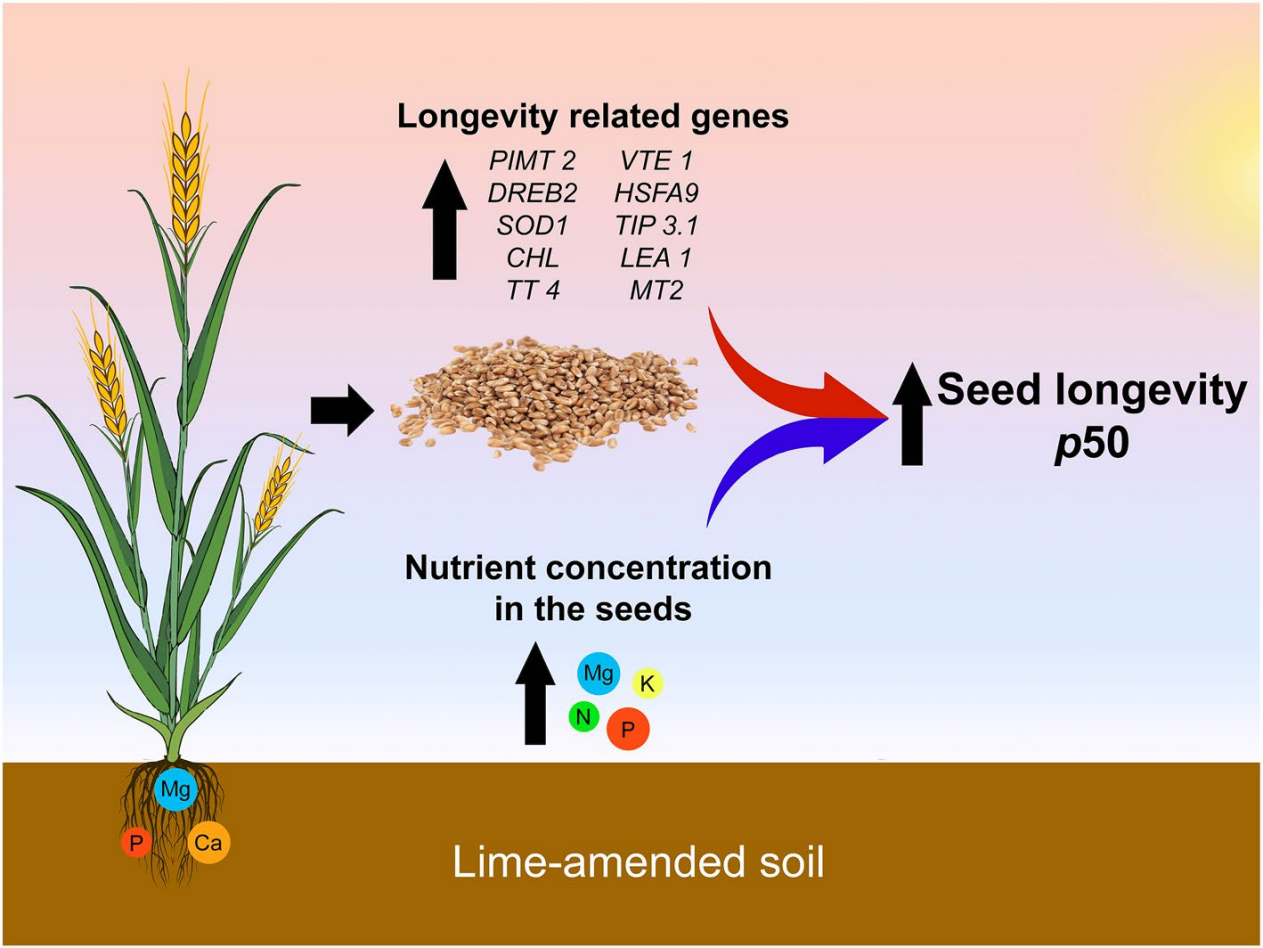
Summarized results of this study representing the cascading effects of improving soil fertility on seed nutrient content, longevity related genes, and *p*50.

As mentioned earlier, the ability to retain viability during storage is associated with protection mechanisms^5^. In order to demonstrate this at gene transcript levels, we studied the expression of genes related to protection mechanisms (Fig. 2) and consequently seed longevity. Our findings show that seeds produced in soil with lime application have a superior quantity of the transcription factors (HSFA9, DREB2), antioxidants (VTE1) and proteins genes (LEA 1, PIMT 2, CHL), hence extending the longevity of wheat seeds (Fig. 4). These results were supported by the correlation studies performed between longevity (*p*50) and gene expression (Fig. 3). In seeds produced in acid soils, this higher expression of genes did not occur, and they showed a faster decline in their longevity (Fig. 1D). The expression of the genes that we selected to study was consistently related to seed longevity^27–31^. Thus, our research showed by physiological assays and gene expression that acid soils impact the storage capacity of wheat seeds, while liming is a practice that mitigates the problem.

Following our investigation, the redundancy analysis revealed that Mg and P were the most important nutrient responsible for the increase in gene expression and longevity (*p*50) in wheat seeds (Fig. 3C). In the absence of lime application, there was restricted availability of Mg and other nutrients (Table 1) for seed filling. Mg is part of the composition of numerous proteins^32,33^ with a potential protective function against oxidative stresses^34^. In our study, an intercorrelation between Mg content, *VTE1* expression and longevity has been demonstrated (Fig. 3D), which allows us to associate the Mg content in the seeds with protection against oxidative damage since the *VTE1* gene prevents lipid peroxidation during storage as demonstrated in *Arabidopsis* seeds and thus, expands its longevity^28^. In addition, Mg deficiency prevents the translocation of photoassimilates over long distances in the plant and reduces its partitioning to seeds^35,36^. Some of these compounds are reserves that protect seeds during desiccation and storage, ensuring their autonomy in the dry state^2,5^. Thus, the idea is that Mg deficiency in acid tropical soils has a negative effect in seed longevity.

Acid soils are naturally less fertile due to limited availability of nutrients, including Mg and P^37^. The application of lime provides greater Mg and P availability to wheat plants (Table 1). In particular, P plays a key energetic role in converting stored seed compounds into new tissue, and enables rapid seedling formation^38^. The concentration of P in seeds determines their vigor through seedling performance, especially root growth^39^. Since seedling emergence capacity is related to seed longevity^40^, a connection can be established between the reasons why phosphorus contributes to superior storage capacity reported in this study (Fig. 3C). These results mentioned earlier are important, regarding what is known in relation to the contribution of the nutrient concentration in seeds and seed storability, because they demonstrate the potential contribution of Mg and P in seed longevity and the preservation of seed vigor during storage (Figs. 1D,E, 3).

Finally, our results support the idea that apparently wheat plants have the ability to adjust their seed production to the availability of soil nutrients without affecting germination and vigor (Fig. 1A,B), as a strategy to ensure their propagation; nevertheless, without ensuring dispersion over time (reduced longevity) (Fig. 1C,D). Therefore, managing soil acidity with lime provides soil fertility improvement and plants with the adequate nutrient status^8^ ensuring production of wheat seeds with superior longevity (Fig. 1). Thus, our work makes it clear that acid soils, among other environment factors already described, have an influence on seed longevity (Fig. 4).

## Conclusions

The application of lime mitigates acidity in tropical soils and ensures the production of seeds with enhanced chemical composition and longer life span.

## Supplementary Information

Supplementary Information 1.

Supplementary Information 2.
