# Supplementary figures and images for: Liming enhances longevity of wheat seeds produced in acid soils
Source: Sci Rep. 2022 Oct 27;12:18035. doi: 10.1038/s41598-022-21176-6 (PMC9613768; doi:10.1038/s41598-022-21176-6)

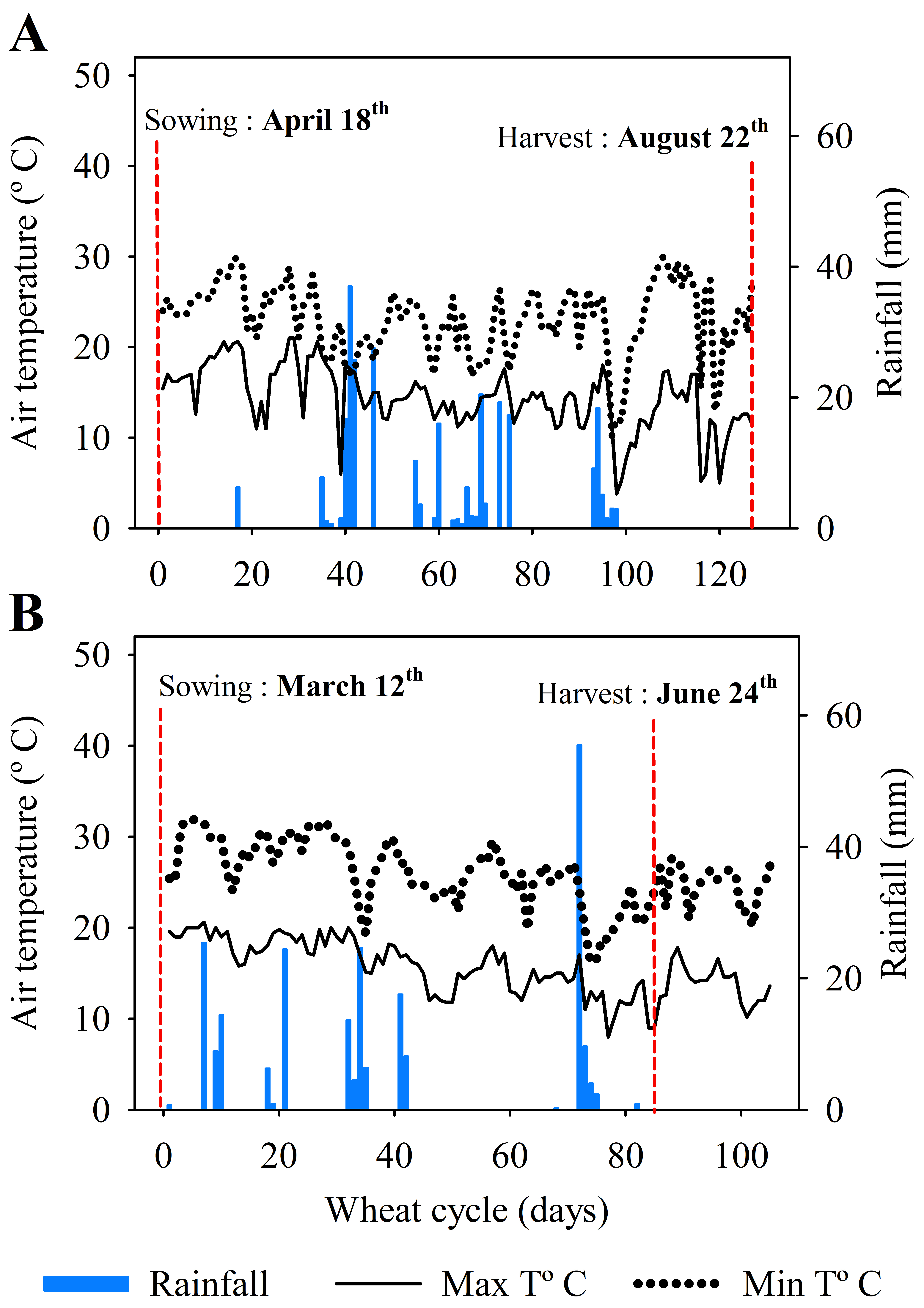

Supplement: Supplementary file 1 — Supplementary Information 1. [file 41598_2022_21176_MOESM1_ESM.jpg]
