# Supplementary material for: Liming enhances longevity of wheat seeds produced in acid soils
Source: Sci Rep. 2022 Oct 27;12:18035. doi: 10.1038/s41598-022-21176-6 (PMC9613768; doi:10.1038/s41598-022-21176-6)
Supplement: Supplementary file 2 — Supplementary Information 2. [file 41598_2022_21176_MOESM2_ESM.docx]

**Supplementary material**

**Table S1.** Previous crops and treatments in the experimental area over twelve years.

| Cropping season | Month | | Crops | Treatments |
| --- | --- | --- | --- | --- |
| 2002/2003 | | Nov | *Oriza sativa* | Liming  [71% ECCE*] |
|  |  | Apr | *Avena strigose* |  |
| 2003/2004 | | Jan | *Phaseolus vulgaris* | - |
|  |  | Apr | *Avena strigose* |  |
| 2004/2005 | | Nov | *Arachis hypogaea* | Liming  [71% ECCE] |
|  |  | Apr | *Avena sativa* |  |
| 2005/2006 | | Nov | *Arachis hypogaea* | - |
|  |  | May | *Avena sativa* |  |
| 2006/2007 | | Feb | *Zea mays* intercropped with *Urochloa brizantha* | - |
|  |  |  | *Urochloa brizantha* |  |
| 2007/2008 | | Dec | *Zea mays* intercropped with *Urochloa brizantha* | - |
|  |  |  | *Urochloa brizantha* |  |
| 2008/2009 | | Dec | *Glycine max* | - |
|  |  | Jun | *Avena strigose* |  |
| 2009/2010 | | Oct | *Glycine max* | - |
|  |  | Mar | *Sorghum vulgare* |  |
| 2010/2011 | | Nov | *Zea mays* | Liming  (88% ECCE) |
|  |  | Apr | *Crambe abyssinica* |  |
|  |  | Sept | *Vigna unguiculata* |  |
| 2011/2012 | | Nov | *Zea mays* | - |
|  |  | May | *Crambe abyssinica* |  |
|  |  | Sept | *Vigna unguiculata* |  |
| 2012/2013 | | Jan | *Pennisetum glaucum* | - |
|  |  | Apr | *Triticum aestivum* |  |
| 2013/2014 | | Nov | *Phaseolus vulgaris* | - |
|  |  | Mar | *Triticum aestivum* |  |

***** Reapplications in 2002, 2004, and 2010 were added to the soil surface when the base saturation reached ≤ 50%. ECCE: Effective calcium carbonate equivalent.

**Table S2.** Calculation of the recommended total rate of dolomitic lime to increase base saturation in the topsoil layer (0.0–0.20 m) to 70%.

| **Equations** | | **Description** |
| --- | --- | --- |
| Eq. 1 | DR (Mg ha^-1^) = CEC × (BS2 - BS1) / (10 × ECCE*) | Where BS2 is the estimated base saturation (70%), and BS1 is the base saturation measured by the soil analysis, as shown in Eq. 2. |
| Eq. 2 | BS1 (%) = (Ca^2+^ + Mg^2+^ + K^+^) × 100/CEC | Where Ca^2+^, Mg^2+^, and K^+^ are basic exchangeable cations (mmol_c_ kg^-1^), and CEC is the total cation exchange capacity, calculated as shown in Eq. 3. |
| Eq. 3. | CEC (mmol_c_ kg^-1^) = Ca^2+^ + Mg^2+^ + K^+^ + (H+Al) | - |

***** ECCE is the effective calcium carbonate equivalent.

**Table S3.** Genes used to design the primers was taken from the NCBI database for *Triticum aestivum* homologous sequences.

| **Gene name** | **Identification** | **Forward primer** | **Reverse primer** |
| --- | --- | --- | --- |
| ABSCISIC ACID INSENSITIVE 3 (ABI3) | FJ640559.1 | GGTGATTTCATCGTGCTTTACTC | TTGTGCTTGGCTAGATCCTG |
| ABSCISIC ACID INSENSITIVE 5 (ABI5) | AB362818.1 | CAGGCTTATACAATGGAGTTGG | GTCCGAACTGATCCTTCATCTC |
| HEAT SHOCK TRANSCRIPTION FACTOR A9 (HSFA9) | KF208543.1 | GAAGAGATTGAGATGCTAAAGAGG | TTGCTCCATTCCGTGAAGAC |
| HEAT SHOCK TRANSCRIPTION FACTOR A4A (HSFA4A) | KF208542.1 | CTGGTCCTGAGTGTATATTCCC | GTCATCGGTCTCATCAGTGTC |
| DROUGHT RESPONSIVE ELEMENT BINDING PROTEIN 2 (DREB2) | AB193608 | TACTACAACGTCCACCAACC | GTAATGCTCGACAGACTCCA |
| LEAFY COTYLEDON 1 (LEC1) | KM078734.1 | TACGGGTACGAGGAAGGAG | GTCATGCGATTCTTCTGCTG |
| VITAMIN E DEFICIENT 1 (VTE1) | DQ456882.1 | CACATCTAAGCAGAAGTCAACAG | CTCCATCCCATTCTATCCATCC |
| ASCORBATE PEROXIDASE 2 (APX2) | EF555121.1 | CAAATGGTTCAATTAGATACGAGG | TGGATGCTTCGCTTTAATAGG |
| SUPEROXIDE DISMUTASE 1 (SOD1) | KP313757.1 | GTTGAAACTACTCCTAATCAGGAC | ATGCTCCCAGACATCAATTCC |
| GLUTATHIONE S-TRANSFERAS (GST) | AJ414700.1 | TACGAGTACGTGGAGGAGAG | TCGATGTACTGCACGATGAC |
| METALLOTHIONEIN 2 (MT2) | AF470355.1 | TAGCAGAGCGGATAAACTCAG | ATCTCAGGGTACATCTTGCAC |
| MERCAPTOPYRUVATE SULFURTRANSFERASE 2 (MST2) | AY036608.1 | CTCACCAAGTAGTTGATGCC | GTATATGCCCACTTCTAACTCC |
| CHLOROPLASTIC LIPOCALIN (CHL) | DQ223009 | TTCACCTGACGGATACATCAC | ACTTGCTGCTGATCATCTCC |
| PROTEIN-L-ISOASPARTATE METHYLTRANSFERASE 2 (PIMT 2) | L07941.1 | ACAGTCCTATGCCTATTGGT | TAATCCTTCAACAGTTCTAAGCAG |
| ALPHA-TONOPLAST INTRINSIC PROTEIN (TIP3,1) | AB535600.1 | CGTGACCGTGAACATCTCC | GCGCGATCCAGTAGAAGAG |
| TRANSPARENT TESTA 4 (TT 4) | AY286098.1 | TTCAAGATCACCAAGAGCGA | CTTCCTGATCTGCGATTTGTC |
| EM6 - LATE EMBRYOGENESIS ABUNDANT 1 (EM6 –LEA 1) | X73229 | GGTACAGCGAGATGGGTC | AGGACTTGGTCTTGAACTTGG |
